# Initial Imaging Analysis of Budd-Chiari Syndrome in Henan Province of China: Most Cases Have Combined Inferior Vena Cava and Hepatic Veins Involvement

**DOI:** 10.1371/journal.pone.0085135

**Published:** 2014-01-08

**Authors:** Pengli Zhou, Jianzhuang Ren, Xinwei Han, Gang Wu, Wenguang Zhang, Pengxu Ding, Yonghua Bi

**Affiliations:** Department of Interventional Radiology, The First Affiliated Hospital, Zhengzhou University, Zhengzhou, Henan Province, China; Yonsei University College of Medicine, Republic of Korea

## Abstract

**Aim:**

To evaluate the type of venous involvement in Chinese Budd-Chiari syndrome (BCS) patients and the relative diagnostic accuracy of the different imaging modalities.

**Methods:**

Using digital subtraction angiography (DSA) as a reference standard, color Doppler ultrasound (CDUS), computed tomography angiography (CTA), and magnetic resonance angiography (MRA) were performed on 338 patients with BCS. We analyzed the course of the main and any accessory hepatic veins (HVs) and the inferior vena cava (IVC) to assess the etiology of obstructed segments and diagnostic accuracy of CDUS, CTA and MRA.

**Results:**

Among the 338 cases, there were 8 cases (2.4%) of isolated IVC membranous obstruction, 45 cases (13.3%) of isolated HV occlusion, and 285 cases (84.3%) with both IVC membranous obstruction and HV occlusion. Comparing with DSA, CDUS, CTA had a diagnostic accuracy of 89.3% and 80.2% in detecting BCS, and 83.4% of cases correctly correlated by MRA.

**Conclusion:**

In Henan Province, most patients with BCS have complex lesions combining IVC and HV involvement. The combination of CDUS and CTA or MRI is useful for diagnosis of BCS and guiding therapy.

## Introduction

Budd-Chiari syndrome (BCS) is a rare disease in Western countries. The prevalence is approximately 1∶100,000, [1] and thrombotic obstruction of hepatic veins (HVs) is the most important cause. [2], [3], [4] One predisposing thrombophilic factor, such as Myeloproliferative neoplasms (MPNs), JAK2 V617F mutation and factor V G1691A mutation (FLVM), can be found in at least 90% of BCS patients, of which, MPNs are the most common cause and account for about 41% of cases. [5], [6], [7] However, the prevalence of BCS is higher in less developed countries, such as China, South Africa, India and Nepal. For example, BCS is more common in areas along the Yellow River and Huaihe River Basin, especially in Henan and Shandong Provinces of China. Unlike the Western countries, it is reported that membranous obstruction of inferior vena cava (IVC) represents the most common etiological factor, and only a few cases have an underlying thrombotic factor. [5], [8], [9] These differences excite wildly interest and need further investigation.

The Henan Province has a population of 130 million, accounting for one-thirteenth of the total population of China, which is also the region with considerably high BCS incidence rate. Unfortunately, there is a lack of knowledge about BCS etiology and its imaging features. Nowadays, research on BCS has been growing, especially with the availability of Doppler ultrasound (US), CT angiography (CTA), magnetic resonance angiography (MRA), and digital subtraction angiography (DSA). [10], [11], [12] This study aims to evaluate the type of venous involvement in Chinese Budd-Chiari syndrome (BCS) patients and the relative diagnostic accuracy of the different imaging modalities.

## Materials and Methods

### Ethics

This study has been approved by The First Affiliated Hospital of Zhengzhou University Ethics Review Committee and the National Ethics Review Committee. Written informed consents were obtained from all patients and the guardians, caretakers, or the next of kin on the behalf of the participants involved in this study.

### Patients

All patients came from the regions along the Yellow and Huaihe Rivers, and most of them lived in rural areas under adverse socioeconomic and public health conditions. From August 2006 to October 2010, 338 BCS patients were admitted to the interventional wards of No.1 Affiliated Hospital of Zhengzhou University.

### Procedures of Doppler US, MRI, CT and DSA

Doppler US was performed on a seven-color apparatus (General Electric, USA), examining the HV and IVC using acoustic windows under the xiphoid and costal margin. A 6-slice spiral CT (Brilliance, Philips, Holland), a 16-slice CT (Lightspeed, General Electric, USA), and 64 rows of CT machines (Lightspeed, General Electric, USA) were used. After acquisition of baseline unenhanced images, a bolus of iodinated contrast material was administered and images of the liver were acquired in the arterial phase (25–30 s after injection), portal venous phase (60 s after injection), and IVC phase (180 s after injection). The IVC phase was timed in order to enable a stenotic IVC to fill with contrast medium. [13] Images were formatted in the axial, sagittal, and coronal planes. MRI with a dynamic enhanced angiography sequence was performed on a 1.5 Tesla or 3.0 Tesla Signa (HDxt 3.0T, General Electronic, USA). Enhanced scanning was commenced 15 s after injecting of contrast agent (0.2–0.4 ml/kg body weight) to obtain dynamic 3D images. By using Shimadzu Digitex (Shimadzu, Tokyo, Japan) equipment, DSA was performed to opacify the IVC via femoral vein injection. Hepatic venous opacification was achieved by retrograde injection or, if necessary, percutaneous transhepatic catheterization. [14].

### Image Evaluation

Doppler US examinations were evaluated by two experienced operators (Ren JZ and Wu G) together, with a third physician (Han XW) required in case of different opinions. Diagnosis was not made until two physicians agreed. All the scanning images were viewed separately by two experienced associate chief physicians (Ren JZ and Wu G), with a third physician (Han XW) required in case of different opinions. All the radiologists diagnosed randomly and respectively, they were blinded to patient information and did not know the results of the DSA images when they are reading the ultrasound, CTA or MRA images.

### Statistical Analysis

All qualitative data were expressed as proportions. The chi-square test was for study the categorical variables (SPSS Inc., Chicago, IL, USA). Differences were considered statistically significant when *P* was less than 0.05.

## Results

There were 209 males and 129 females, ranging from 12 to 72 years old, with an average age of 41.7±10.1 years. The imaging examinations were completed within one week for patients suspected by clinical symptoms. All patients were firstly scanned by CDUS, CTA and/or MRA were further performed if necessary. All BCS cases underwent DSA to confirm the diagnosis of BCS during radiological intervention. Fifty-five cases were confirmed by CDUS, 126 cases by CDUS and CTA, and 157 cases by CDUS and MRA.

### Evaluation of CDUS, MRI, CT and DSA

Comparing with DSA, CDUS had a diagnostic accuracy of 89.3% (302/338) in detecting BCS, with a 3.6% (12/338) of false-positive diagnoses of BCS and 7.1% (14/338) of false-negative. Among 126 cases confirmed by Doppler US and CTA, CTA has an overall accuracy of 80.2% (101/126), with a 6.3% (8/126) of false-positive and 13.5% (17/126) of cases indeterminate when compared with DSA findings. Among 157 patients studied by MRA, 83.4% (131/157) of cases correctly correlated with DSA findings. The false-positive and false-negative were 11.5% (18/157) and 5.1% (8/157), respectively.

**Figure 1:**
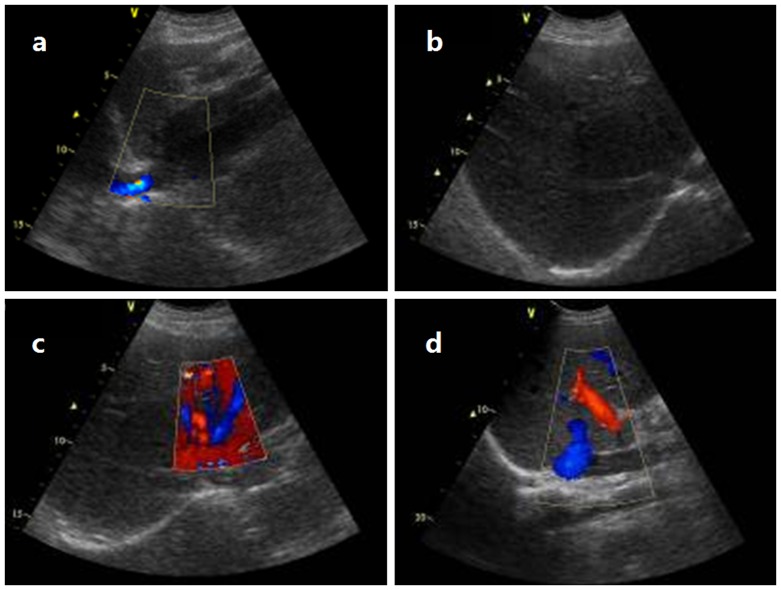
The preoperative color Doppler flow imaging. CDUS shows a membranous obstruction of IVC wiht thin beam-like flow (Figure 1 a), and the right HV is completely obstructed (Figure 1 b); There is a obliteration after the confluence of left HVs without blood flows (Figure 1 c), the right HV has compensatory enlargement, with blood flow draining into IVC (Figure 1 d).

**Figure 2:**
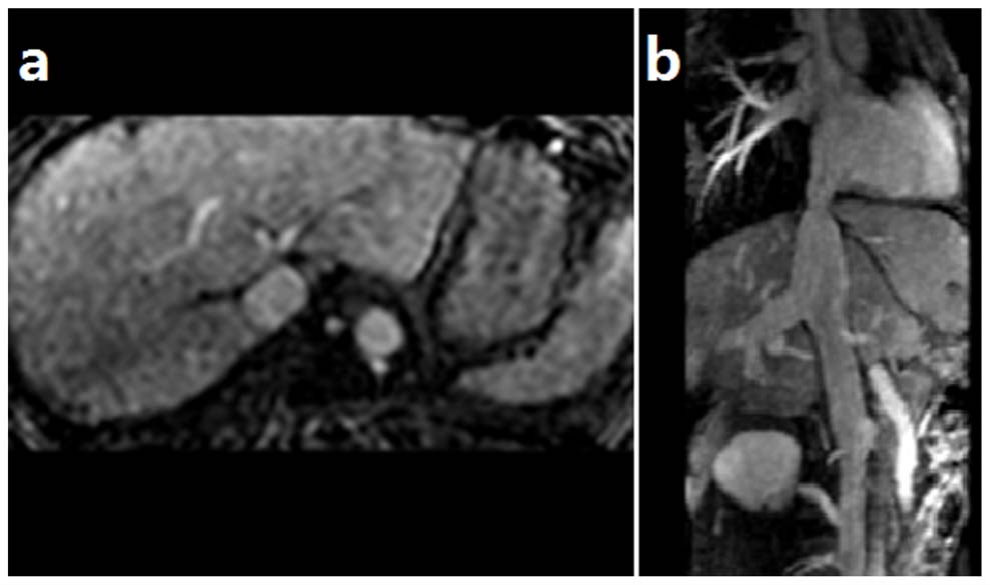
MRI axial and 3D reconstruction images for showing HVs and IVC. The right HV is completely obstructed and the middle and left HVs are obstructed after their confluence (Figure 2 a). The IVC shows a membranous stenosis, and the right HV is compensatory enlarged (Figure 2 b).

**Figure 3:**
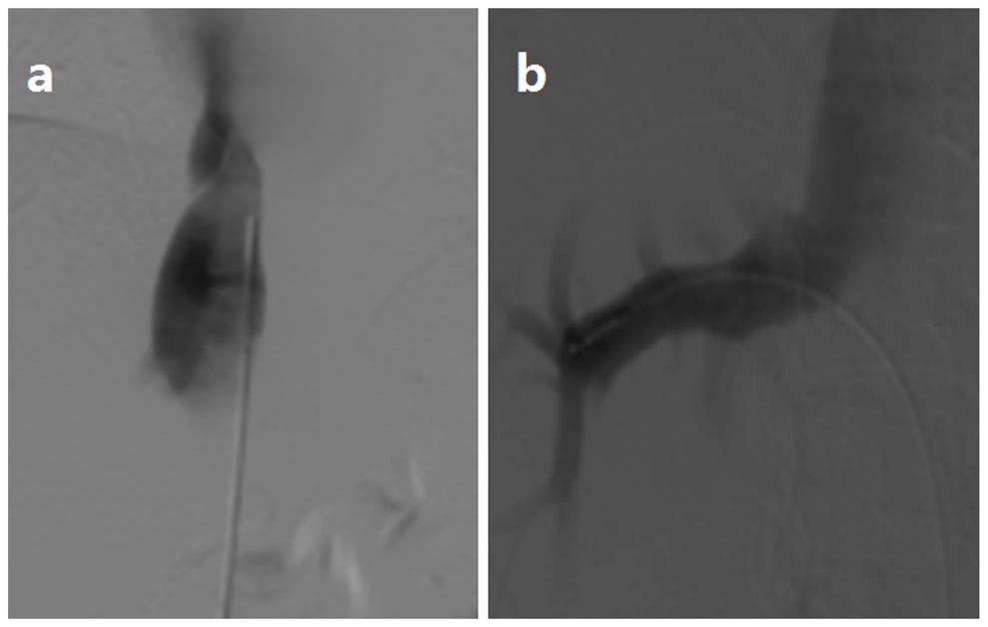
The inferior venocavography through the femoral access. The IVC is obstructed at the secondary porta of liver, and the adopted contrast agent is thin and beam-like (Figure 3 a), the right and rear HVs are remarkably dilated (Figure 3 b).

**Figure 4:**
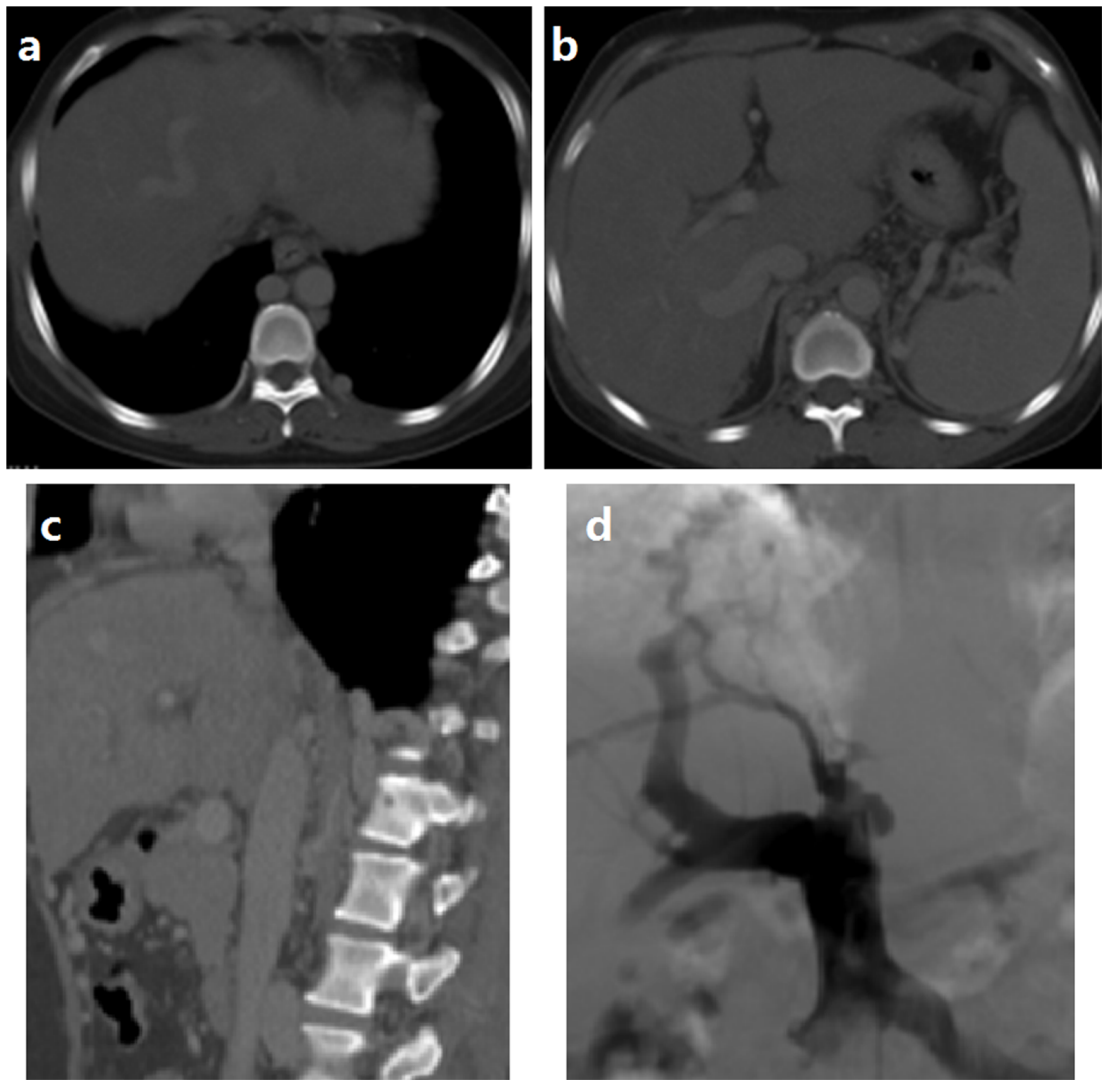
The CTA images of portal venous phase and DSA image during intervention. The middle HV is obstructed, the right and left HVs are completely obstructed (Figure 4 a); The right, rear and inferior HVs are expanded (Figure 4 b), and the segmental IVC is obstructed (Figure 4 c); DSA image shows a segmental obstruction of the inferior caval vein, and intrahepatic collateral circulation for drainage of HV flows (Figure 4 d).

CDUS is sensitive for membranous obstruction of IVC. HVs and IVC were devoid of flow signal once completely obstructed. HV may compensatory enlarge (Figure 1). Turbulent, reversed or stagnant flow in HVs can be easily depicted by CDUS. Heterogeneous patchy enhancement, nonvisualization of HVs, intrahepatic collaterals and obstruction of the IVC are important findings of CT or MRI. MRI allows good anatomic orientation of HVs or IVC due to its multiplanar capacity, and IVC can be better visualized on coronal images (Figure 2). DSA are useful for showing the level and extent of obstruction (Figure 3). The CT examination results were consistent with those of DSA (Figure 4).

### Focal Liver Abnormality

68.6% (232/338) of cases were of hepatic cirrhosis, and ascites were often found during image assessment. Intrahepatic collateral circulation and collateral circulation in the body were identified in 62.1% (210/338) and 65.1% (220/338) of cases due to portal hypertension. 48.2% (163/338) of those patients had wider accessory HVs or large HV collaterals. About half of cases showed caudate lobe enlargement, the caudate lobe hypertrophied and reached a mean diameter of 33.1±3.2 mm. Benign regenerative nodules were found in 23.4% (79/338) of cases, which were typically small and multiple, with hypervascularity during the arterial and portal venous phases. Hepatocellular carcinoma was found in 7.7% (26/338) of patients. For identifying parenchymal abnormalities, CT and MRI had a higher sensitive modality than CDUS (90.5%, 91.3% versus 76.8%, *P*<0.05).

### Type Analysis of BCS

Out of 338 patients with BCS, 293 cases (86.7%) showed IVC involvement, in which, 285 cases had an associated HV obstruction (84.3%), and 8 cases had isolated IVC involvement (2.4%). Of 330 cases with HV involvement (97.6%), 45 cases (13.3%) had isolated HV obstruction with a normal IVC. Among 285 patients with venous lesions involving both the IVC and HV, there were 186 cases (55.0%) with obstruction of the left, middle and right HV branches and 82 cases (24.3%) with obstruction of two branches (77 cases were obstructed in the left and middle branches; 2 cases in the left and right branches; and 3 cases in the middle and right branches), the remaining 17 cases (5.0%) displayed obstruction of one HV branch, of which 11 cases had obstruction of the right HV, 3 cases of the middle HV, and 3 cases of the left HV. IVC lesions included 200 cases (59.2%) of complete occlusion and 93 cases (27.5%) of stenosis (Table 1). Of the 200 occlusive cases, there were 163 cases (48.2%) of membranous obstruction and 37 cases (10.9%) of thrombotic obstruction of the IVC. 209 cases (61.8%) were membranous obstruction if adds the stenotic cases caused by membrane in IVC.

**Table 1:** Types distribution of venous lesions in patients with BCS.

Lesion types	Cases	Proportion (%)
**IVC and HV involvement**	285	84.3
Obstruction of three main HVs	186	55.0
Obstruction of two main HVs	82	24.3
Obstruction of one HV	17	5.0
**Isolated HV involvement**	45	13.3
Obstruction of three main HVs	44	13.0
Obstruction of two main HVs	0	0.3
Obstruction of one HV	1	2.2
**Isolated IVC involvement**	8	2.4
Occlusive IVC	6	1.8
Stenotic IVC	2	0.6

IVC: inferior vena cava; HV: hepatic vein.

Among 45 patients with isolated HV obstructive lesions (Table 1), there were 44 cases of involving the left, middle, and right HVs, and one case with the obstruction of only the right HV. Among 8 patients with isolated IVC obstructive lesions, 6 cases were occlusive and 2 cases were stenotic. All of 8 cases were of the membranous type. There was no significant difference among the findings of CDUS, CTA, MRA, or DSA according to the chi-square test (*P*>0.05).

## Discussion

Thrombotic obstruction of HVs is considered as the most important cause of BCS in European regions. [2], [3], [4] The predisposing thrombophilic factors, particularly thrombosis due to the hypercoagulable state, or thrombophlebitis resulted from long-term oral contraceptives, cause the secondary onset of thrombus in HV and obstruction of HV. [15] In the Eastern countries, Africa and other less developed countries, BCS was considered to be due to IVC membranous obstruction in the rear segment of the liver, rarely due to thrombosis, and there was no intravasculitis, hypercoagulability or thrombosis, [16] which was once called IVC diaphragm obstructive syndrome. In 1962, Kimura initially conducted the IVC diaphragm intervention through the heart. [17] More cases were then reported in Japan, India, Asia, and Africa. China is one of the areas suffering from a high incidence of BCS, and Chinese BCS is generally considered as one of the subtypes. The type of BCS in the West is quite different from that of less developed regions in the East. Among 338 cases of BCS confirmed by various imaging modalities, the most common type had involved both the IVC and HV, accounting for 84.3% of all cases. This result suggests that IVC and HV involvement is the predominant pattern of Chinese BCS, or at least in Henan Province, which is consistent with the relevant reports in recent years. Similarly, Cheng D et al. reported that 61% of cases are membranous obstruction, only 5% of cases have MPNs with a JAK2 V617F mutation, and most of BCS patients (63%) suffered from both HVs and IVC obstruction. [9].

As for the reason, inherited predisposition and acquired thrombogenic stimulus may converge in the pathogenesis of BCS. [18], [19] FLVM is quite common in Caucasian populationsbe and is found in at least 90% of BCS patient, interestingly, this mutated allele is not determined in Eastern populations, which indicates that genetics differences between Western and Eastern populations should account for the discordance of BCS. [20] The most patients are chornic BCS and the course of disease is quite long in China and Japan. [9], [21] The situation in the East is different from that in the West where most patients are acute BCS, and the type of lesion may vary according to geography. [9].

BCS is distributed regionally in China, and there is the highest prevalence in the Huanghuai region. [22] The most prevalent etiological factor of BCS is also membranous obstruction in China. Females of childbearing age are more likely to develop BCS in Western countries, however, males with the median age of 46 years account for the majority of cases, and most of cases are chronic BCS. [9] The HVs obstruction is lower, but the IVC obstruction rate in Chinese patients is far higher when compared with those in Western countries, indicating that BCS may vary from region to region. In Henan Province, more than 80% of BCS cases are peasant, and people in countryside are more likely to develop BCS than oppidan in city. These indicate that BCS is related to environmental conditions, diet and economic factors to some extent in Henan Province, or even in China.

Various imaging modalities are available for dignosing BCS in the clinical setting, including CDUS, CT, MRI and conventional venography. The CDUS should be performed as the first imaging method of choice in BCS due to its high diagnostic sensitivity, inexpensiveness and convince, without contrast material. [23], [24] The CDUS has a diagnostic sensitivity of 75.0% to 90%. [23], [24], [25] HVs and IVC devoid of flow signal and collateral hepatic venous circulation can be indicative of BCS. [26] Echogenic membrane can be easily depicted by sonography. However, sonography is operator-dependent and interfered with intestinal gas and excessive ascites. [27].

The second line of investigation should be CT or MRI. Heterogeneous patchy enhancement, nonvisualization of hepatic veins, intrahepatic collaterals and obstruction of the IVC are their important findings. For identifying the status of hepatic venous thrombosis, CT has an overall accuracy of 50%. [28], [29] CT has a higher sensitive modality than sonography (90.0% versus 70.0%) in showing parenchymal abnormalities. [28] Our results confirmed those findings. Moreover, CTA is useful in investigating the patency of TIPS shunt and vascular involvement. [27] The limitations of CT are the possible allergic reaction and nephrotoxicity due to the use of contrast material, and CT is often unable to show web in IVC.

MRI allows good anatomic orientation of HVs or IVC due to its multiplanar capacity. The status of 80.0% of cases correctly correlated with pathology when using MRI. [28] The advantages of MRA over CDUS include better anatomic orientation, short examination time without operator-dependency or restriction from intestinal gas. [27] MRI is also advantageous over CT imaging in some aspects. [27] The combination of CDUS and CT or MRI is useful for guiding therapy due to the optimal delineation of HV and IVC obstruction. [26].

Hepatic venography and inferior venocavography are conventional diagnostic method for BCS, which currently serve as a third line of investigation. Venography combined with venous pressure measurements should be performed when radiological intervention or surgical shunting is considered. [25], [26] However, venography often requires cannulation of HVs and large amounts of contrast medium. [26].

Most of Chinese patients are chronic, ascites, caudate lobe enlargement and abnormal hepatic configuration are present in vast majority of the BCS cases. [27] Half of patients have wider accessory HVs or large HV collaterals, which can help to drain off the blocked HVs blood to relieve portal hypertension. [9] Caudate lobe may hypertrophy due to its separate drainage into the IVC. [30] Contrast-enhanced CT reveals inhomogeneous enhancement of liver, such as peripheral regions with low attenuation due to portal hypoperfusion and focal enhancing area owning to arterio-portal shunting. [30] Benign regenerative nodules, intrahepatic collateral circulation, body collateral circulation and hepatic cirrhosis can be seen in chronic BCS cases. [31] Hepatocellular carcinoma often develops in chronic end-stage liver disease, [27], [31] sometimes it will be shown in BCS cases.

Knowledge of the morphologic type of BCS is useful for guiding the therapeutic approach and assessing operation risk. Shunt surgery is no longer the standard care for BCS, with advances in radiological interventions and a good midterm outcome. [32] Interventional techniques can aid in treating and stabilising the patient for possible orthotopic liver transplantation when liver dysfunction progressive. [33].

Percutaneous transluminal angioplasty (PTA) can be used for patients with post-hepatic IVC membranous-type obstruction, balloon dilatation and percutaneous stent placement have been found to be the techniques for those with segmental IVC obstruction [34] or IVC web [35]. PTA is seldom adopted in Western countries, however, and stenting are effective and safe for treating BCS, with a good long-term outcome, [36] because most chronic BCS are caused by membranous obstruction. According to our experiences, large balloon dilation with a diameter of 25–30 mm ballon catheter is safe and effective for most IVC obstruction cases, and only few cases need further stenting after large balloon dilation. The blood backflow of HV and liver function can be basically compensated, as long as there was a widely patent HV, regardless of which branch of the main HVs was or whether it was the accessory HV. PTA, mainly balloon dilation, is suitable for this kind of BCS. TIPS and orthotopic liver transplantation are usually adopted in Western countries, although the survival rate is poor. [37].

In case of short segment occlusion or stenosis of HV and/or IVC, the obstruction between remnant HV and IVC should be reopened by means of balloon angioplasty with or without stent placement. [38] TIPS and surgical options are reserved for patients with failure of interventions due to complete occlusion of HV. [32] Patients with both the and segmental IVC occlusion require opening of both occlusive segments, these may be difficult and challenging and the several multiple approaches should be required. [32] The IVC occlusion should be reopened as long as they have unobstructed HV blood flow. [34] Besides, additional therapies with anticoagulation, pharmacological or endoscopic treatment for variceal bleeding and diuretics for ascites should be considered. [26].
